# The Diagnostic and Prognostic Value of Neutrophil-to-Lymphocyte and Platelet-to-Lymphocyte Ratios in Urosepsis

**DOI:** 10.3390/medicina61091713

**Published:** 2025-09-19

**Authors:** Petru Octavian Drăgoescu, Bianca Liana Grigorescu, Andreea Doriana Stănculescu, Andrei Pănuș, Nicolae Dan Florescu, Monica Cara, Maria Andrei, Mihai Radu, George Mitroi, Alice Nicoleta Drăgoescu

**Affiliations:** 1 Department of Urology, Faculty of Medicine, University of Medicine and Pharmacy of Craiova, 200349 Craiova, Romania; octavian.dragoescu@umfcv.ro (P.O.D.); andrei.panus@umfcv.ro (A.P.); mihai.radu@umfcv.ro (M.R.); george.mitroi@umfcv.ro (G.M.); 2 Department of Anesthesiology and Intensive Care, George Emil Palade University of Medicine, Pharmacy, Science, and Technology of Targu Mures, 540142 Targu Mures, Romania; bianca.grigorescu@umfst.ro; 3 Department of Anesthesiology and Intensive Care, Faculty of Medicine, University of Medicine and Pharmacy of Craiova, 200349 Craiova, Romania; 4 Department of Gastroenterology and Hepatology, University of Medicine and Pharmacy of Craiova, 200349 Craiova, Romania; nicolae.florescu@umfcv.ro; 5 Department of Public Health and Management, University of Medicine and Pharmacy of Craiova, 200349 Craiova, Romania; monica.cara@umfcv.ro; 6 Department of Anesthesiology and Intensive Care, Emergency County Hospital of Craiova, 200642 Craiova, Romania; andreimaria92@gmail.com

**Keywords:** urosepsis, septic shock, neutrophil-to-lymphocyte ratio (NLR), platelet-to-lymphocyte ratio (PLR)

## Abstract

*Background and Objectives:* The severe systemic response to urinary tract infections known as urosepsis is associated with significant morbidity and mortality rates. The neutrophil-to-lymphocyte ratio (NLR) and the platelet-to-lymphocyte ratio (PLR) are simple blood tests that could be useful in predicting the outcome of sepsis. *Materials and Methods:* A prospective observational study was conducted at a tertiary care hospital, where our team studied 223 patients with urosepsis. The patients underwent Sepsis-3 criteria-based urosepsis and septic shock stratification followed by survivor and non-survivor classification. Clinical scores (Sequential Organ Failure Assessment-SOFA, National Early Warning Score-NEWS), laboratory markers (NLR, PLR, PCT-procalcitonin), and patient outcomes were then analysed. *Results:* An admission NLR ≥ 13 was a strong predictor of septic shock (adjusted Odds Ratio (OR) 2.10, 95% Confidence Interval (CI) 1.25–3.54) and in-hospital mortality (adjusted OR 2.45, 95% CI 1.40–4.28). While the prognostic value of the PLR remained moderate, the NLR demonstrated superior predictive power. As easily measurable biomarkers, the NLR and PLR provide valuable information to help clinicians identify at-risk patients during the early stages of urosepsis. *Conclusions:* The NLR is an independent predictor with high predictive value for both septic shock and mortality, performing as well as established clinical scores. The combination of these parameters with clinical assessments could lead to better early decisions and improved outcomes for patients with urosepsis.

## 1. Introduction

Urosepsis is a severe urinary tract infection usually associated with urinary tract obstruction, leading to systemic inflammation that may result in serious consequences. Its early recognition and risk stratification are essential, as progression to septic shock is associated with a marked increase in mortality. While scoring systems—such as Sequential Organ Failure Assessment (SOFA) and National Early Warning Score (NEWS)—and biomarkers—such as procalcitonin (PCT) and lactate—are widely used, they require laboratory infrastructure or clinical assessments that may not always be immediately available in the emergency setting. The neutrophil-to-lymphocyte ratio (NLR) and platelet-to-lymphocyte ratio (PLR) are simple indices derived from complete blood counts, reflecting systemic inflammation and immune dysregulation. Recent research has shown that septic patients with elevated NLR values have more severe disease progression and higher mortality rates. For example, Karamouzos et al. [1] presented evidence that higher NLR values are linked to increased hospital death rates among patients with sepsis, thus establishing its prognostic value; this idea was subsequently confirmed by other studies [2,3]. The PLR serves as an inflammatory marker that reflects the relationship between platelet activation and lymphocyte activity during systemic inflammation. Wang et al. conducted a systematic review and meta-analysis demonstrating that elevated PLR values are strongly associated with higher mortality rates in septic patients, highlighting its potential as a prognostic biomarker [4]. While the prognostic value of the NLR and PLR in general sepsis cases has been studied, their utility in urosepsis cases remains poorly understood. Urosepsis represents a distinct clinical entity often arising in patients with advanced age and multiple comorbidities, in the context of which rapid identification of high-risk patients is crucial for guiding timely interventions. Despite its frequency, few studies have systematically evaluated the NLR and PLR in urosepsis patients or compared their performance with established clinical scores and biomarkers. Investigation of the NLR, PLR, and their relationships with patient outcomes in urosepsis may improve early risk assessment methods and enable better treatment choices. Our study aims to analyse the clinical potential of the NLR and PLR, as simple and affordable biomarkers, for urosepsis risk assessment by examining their predictive value in evaluating the severity of the disease and the clinical outcome of patients with urosepsis.

## 2. Materials and Methods

A single-centre prospective observational study was conducted between 2023 and 2024 in the Intensive Care and Urology Departments of the Craiova Clinical Emergency Hospital. In our assessment, we included 223 patients diagnosed with urosepsis using the Sepsis-3 criteria. Urosepsis was therefore diagnosed in patients with documented or suspected urinary tract infection associated with an elevated Sequential Organ Failure Assessment (SOFA) score (i.e., value ≥ 2), while septic shock was diagnosed in those patients with the need of vasopressor therapy to maintain their mean arterial pressure (MAP) ≥ 65 mmHg and lactate level ≥ 2 mmol/L (18 mg/L) with adequate fluid resuscitation [5]. Prior to study enrolment, signed informed consent was obtained from either the patients or a legal representative (for unconscious patients or those unable to sign). The study was conducted in accordance with the principles of the Declaration of Helsinki after approval from the ethics committee was obtained (approval no. 79/07.04.2023). All patient data were kept confidential, with no patient names or identities recorded. Study enrolment was performed based on certain criteria, including patient age above 18, admitted with urological conditions (e.g., severe urinary tract infections (UTIs), urinary tract obstruction, lithiasis, urinary catheters, or recent urological surgical procedure), and fulfilling the criteria for diagnosis of sepsis. Patients who were younger than 18, immunocompromised, with advanced cancers or other terminal conditions, or pregnant were excluded from the study.

**Figure 1:**
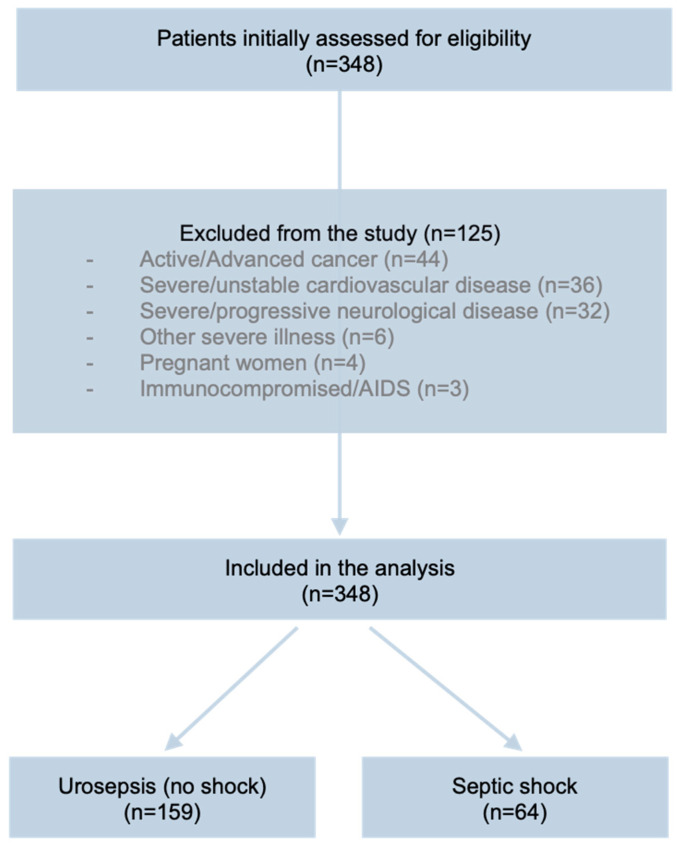
Flow diagram showing the distribution of screened patients, study exclusion criteria, and number of patients enrolled into the study groups.

Depending on the presence or absence of the above criteria, the 223 patients were divided into two study groups (Figure 1): Septic Shock (64 patients) and Urosepsis (159 patients). Following enrolment, we obtained the demographic details and medical history of patients; collected their disease signs, symptoms, and vital signs; and performed a complete clinical examination. We collected information on the patients’ comorbidities according to their medical history and ongoing treatment at the hospital. The main comorbidities included diabetes mellitus, chronic kidney disease, ischaemic heart disease, chronic obstructive pulmonary disease, prior or active malignancy, chronic liver disease, cerebrovascular disease, and dementia.

Routine blood tests (haematology, biochemistry, coagulation, and urinalysis), inflammation markers (C-reactive protein, CRP; procalcitonin, PCT; and lactate), and microbiological samples (urine culture and blood culture—aerobic and anaerobic) were collected at the time of initial evaluation at the hospital’s Emergency Department, prior to the initiation of antibiotic therapy and before any surgical or interventional procedure prior to initiation of treatment. Abdominal ultrasound was performed as an initial imaging assessment for all patients, followed by abdominopelvic computed tomography (CT scan) or other imaging tests according to a patient’s specific requirements. Most of the patients underwent emergency urological procedures for urinary tract drainage (percutaneous nephrostomy, ureteral catheterisation, surgical drainage, or Foley catheter placement). Broad-spectrum empiric antibiotic therapy was initiated upon suspected diagnosis, as well as fluid replacement with 30 mL/kg crystalloid solutions according to the Sepsis-3 bundle recommendations [5]. Patients diagnosed with septic shock despite adequate fluid replacement required additional vasopressor support to maintain MAP above 65 mmHg. Antibiotic therapy was adjusted if needed, according to the urine or blood culture results or the patient’s clinical evolution.

The neutrophil-to-lymphocyte ratio (NLR), the platelet-to-lymphocyte ratio (PLR), and the NEWS and SOFA scores were calculated after enrolment according to the blood test results. Normal NLR values were considered between 1 and 3 [6], while the normal range of the PLR is 90–210 [7,8]. CRP levels were assessed using the standard immuno-turbidimetry method, and PCT was measured using the electro-chemiluminescence immunoassay (ECLIA) method.

All patient information, including demographic, clinical, laboratory, and disease outcome data, was collected from their medical records and lab test results. The first step involved calculating frequency distributions of patient characteristics together with descriptive statistics. The Kolmogorov–Smirnov test was performed to determine the normality of data. Parameter values are presented as mean ± standard deviation (SD) or median and interquartile range for normally distributed data, while categorical values are presented as ratios. The Student *t*-test was employed for normally distributed data, while the Mann–Whitney U-test was used for non-parametric analysis. The Chi-squared test was performed for comparison of categorical variables. The prognostic value of the NLR and PLR in urosepsis was assessed via receiver operating characteristic (ROC) curve analysis to calculate the area under the curve (AUC), with values ranging from 0 to 1 and 0.5 considered as the significant threshold. DeLong’s statistical method was used for multiple ROC curve comparisons. The significance level was established at a minimum of 0.05 for all statistical tests. Through power analysis for ROC curve discrimination (α = 0.05, power = 0.80, null AUC = 0.5) an adequate sample size between 130 and 160 patients was estimated, consequently confirming that our final sample of 223 patients (64 with septic shock, 43 deceased) was acceptable. We also performed multivariate logistic regression analysis to check whether the NLR and PLR are independent predictors of severity (septic shock) and outcome (death). All statistical tests were performed using the licensed MedCalc statistical software, version 23.0 (MedCalc Software, Ostend, Belgium).

## 3. Results

A total of 348 potentially eligible candidates were assessed for study eligibility during the enrolment period. We excluded 125 patients due to various reasons: active or advanced stage cancer (44 cases), severe or unstable cardiovascular disease (36 cases), severe/progressive neurological conditions (32 cases), other severe illnesses (6 cases), pregnant women (4 cases), and AIDS (3 cases). Therefore, 223 patients were finally included into the two study groups, comprising 159 patients with urosepsis and 64 patients with septic shock as detailed in Table 1. We recorded 43 deaths (19.3% average mortality)—18 in the urosepsis group (11.3% mortality) and 25 in the septic shock group (39.1% mortality). The average age of patients was 65.1 ± 13.2 years, with no significant differences observed between the study groups. Most of the patients were males (63.2%, 141 cases) with a 1.7 male/female sex ratio and similar distribution between the study groups. Neither the age or sex distribution between the two groups showed a statistical difference (*p* = 0.156 and *p* = 0.652, respectively). Patients with septic shock had significantly higher SOFA (median 8 vs. 5) and NEWS (median 9 vs. 5) score values and lower GCS scores (median 11 vs. 13) (all *p* < 0.001). As expected, the septic shock group had lower mean arterial pressure values (64.1 ± 5.3 mmHg vs. 78.3 ± 7.7 mmHg, *p* < 0.001) and higher heart and respiratory rates (*p* < 0.001 for both). The septic shock group had significantly higher inflammatory markers, in terms of both the neutrophil-to-lymphocyte ratio (NLR) and platelet-to-lymphocyte ratio (PLR) (NLR: 15 vs. 11; PLR: 268 vs. 173; *p* < 0.001 for both). The septic shock group presented higher lactate, creatinine, bilirubin, and procalcitonin levels, indicating more severe metabolic impairment and organ dysfunction. The results for the two groups showed that there were no significant differences in traditional inflammation markers such as white blood cells (WBC), neutrophils (NEU), lymphocytes (LYM), platelet (PLT), and C-reactive protein (CRP) (*p* > 0.05), indicating that NLR, PLR, lactate, and procalcitonin may provide better discriminatory power for the diagnosis of septic shock.

We further analysed the distribution of the most important comorbidities in our patients, as shown in Table 2. The most common associated conditions within the study population included diabetes mellitus (32.3%), chronic kidney disease (25.1%), ischaemic heart disease (21.5%), chronic obstructive pulmonary disease (COPD) (15.2%), and malignancy (13.0%). Less common comorbidities included chronic liver disease (5.4%), cerebrovascular disease (8.1%), and dementia (4.5%). There were no statistically significant differences in the prevalence of any comorbidity between patients with urosepsis and those with septic shock.

The demographic characteristics and clinical and laboratory;results of 180 survivors and 43 deceased patients were then analysed, as detailed in Table 3. The groups showed no significant age-related differences (*p* = 0.253) or sex-related differences (*p* = 0.652) in terms of mortality. The deceased patients showed higher SOFA (*p* < 0.01) and NEWS (*p* < 0.001) scores and lower GCS scores (*p* < 0.05), confirming the greater severity of disease in these patients. Haemodynamic parameters revealed that deceased patients presented lower MAP values (70.3 ± 10.7 mmHg vs. 75.1 ± 9.1 mmHg, *p* < 0.01) and elevated respiratory rates (26 ± 9 vs. 23 ± 6 breaths/min, *p* < 0.05), but their heart rates remained similar (*p* = 0.325). The deceased patients showed elevated leukocytes (*p* < 0.05) and neutrophils (*p* < 0.05), while lymphocyte and platelet levels were similar between groups. The procalcitonin levels in deceased patients were significantly higher than those in survivors (12.6 [10.6–18.4] ng/mL vs. 11.7 [8.8–14.3] ng/mL, *p* < 0.05), indicating its potential utility for prognosis. The deceased patients showed significantly lower bilirubin levels (*p* < 0.05), while their creatinine and lactate levels remained similar to those in survivors (*p* > 0.05).

The neutrophil-to-lymphocyte ratio (NLR) and platelet-to-lymphocyte ratio (PLR) demonstrated significant associations with patient outcomes among the studied parameters. The deceased patients showed significantly higher NLR and PLR values than survivors (NLR: 16 [13–22] vs. 11 [8–14], *p* < 0.001; PLR: 265 [197–347] vs. 181 [137–265], *p* < 0.001). The elevated NLR and PLR values indicate that patients with urosepsis who experience worse outcomes show signs of systemic inflammation and immune dysregulation. The deceased patients demonstrated higher SOFA and NEWS scores and lower MAP, along with elevated procalcitonin levels, when compared to survivors. In contrast, traditional markers such as WBC, creatinine, and lactate showed less consistent associations with mortality.

**Figure 2:**
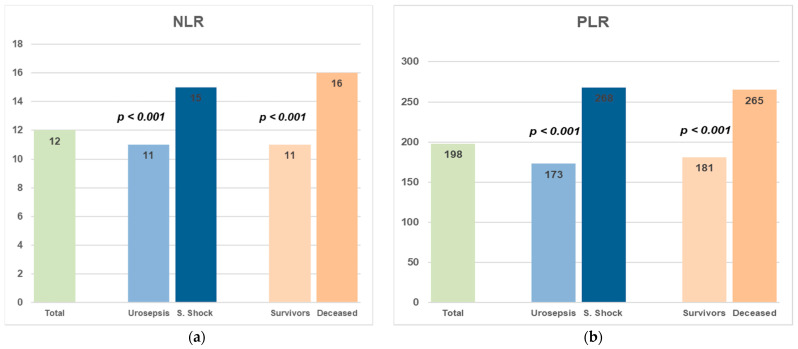
Median values of the Neutrophil-to-Lymphocyte Ratio (NLR) (**a**) and Platelet-to-Lymphocyte Ratio (PLR) (**b**) demonstrate elevated levels in patients with septic shock (S. Shock) and deceased patients for both markers (*p* < 0.001 for both comparisons).

A comparison of the neutrophil-to-lymphocyte ratio (NLR) and platelet-to-lymphocyte ratio (PLR) values between different clinical groups is shown in Figure 2. The median NLR value in the entire population was 12. Patients with septic shock presented higher NLR values (15) than those with urosepsis without shock (11, *p* < 0.001), and the NLR values were significantly higher in non-surviving patients (16) when compared to survivors (11, *p* < 0.001). These results indicate that an elevated NLR is associated with both the severity of infection and mortality in patients. Similarly, PLR values were significantly higher in patients with septic shock and in those who did not survive. The median PLR increased from 173 in the urosepsis group to 268 in the septic shock group (*p* < 0.001) and from 181 in survivors to 265 in non-survivors (*p* < 0.001), as shown below. These patterns indicate a strong relationship between elevated PLR and both disease severity and mortality. The median PLR in the entire cohort was 198. This finding suggests that PLR could be a useful and accessible inflammatory marker for risk stratification in patients with urosepsis.

**Figure 3:**
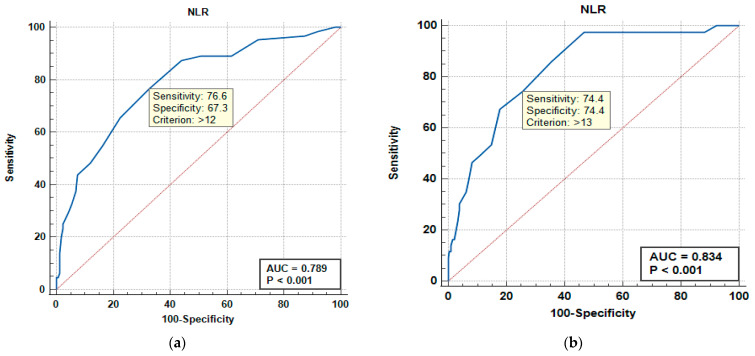
Receiver operating characteristic (ROC) curves showing the performance of the Neutrophil-to-Lymphocyte Ratio (NLR) in predicting septic shock (**a**) and urosepsis-related mortality (**b**). The area under the curve (AUC) values are 0.789 (95% CI: 0.730–0.841) for shock and 0.834 (95% CI: 0.770–0.880) for mortality, indicating very good prediction accuracy for both (*p* < 0.001). The dotted line represents the line of no discrimination (AUC = 0.5).

**Figure 4:**
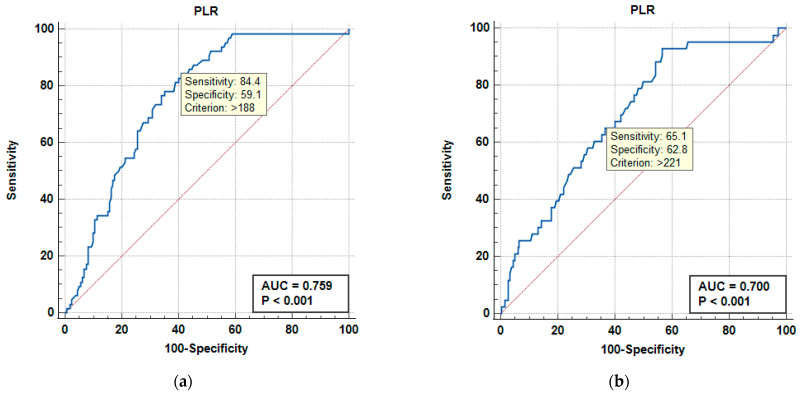
Receiver operating characteristic (ROC) curves showing the performance of the Platelet-to-Lymphocyte Ratio (PLR) in predicting septic shock (**a**) and urosepsis-related mortality (**b**). The area under the curve (AUC) values are 0.759 (95% CI: 0.698–0.814) for shock and 0.700 (95% CI: 0.636–0.760) for death, indicating very good detection accuracy in both cases (*p* < 0.001). The dotted line represents the line of no discrimination (AUC = 0.5).

We continued our assessment of the diagnostic utility of the NLR and PLR using receiver operating characteristic (ROC) curves (Figure 3 and Figure 4) for severity of urosepsis and urosepsis-related mortality. The area under the ROC curve (AUC) with 95% CI was evaluated and the optimal cut-off point was determined via the Youden index, thus providing sensitivity and specificity rates. The NLR showed good predictive ability for severity (septic shock), with an AUC of 0.789 for a cut-off value of 12 (yielding a sensitivity of 76.6% and specificity of 67.3%), and even better predictive ability for mortality at a cut-off value of 13 (with sensitivity and specificity of 74.4% and an AUC of 0.834; *p* < 0.001). The PLR demonstrated similar prediction performance for severity (AUC 0.759, cut-off value 188, good sensitivity of 84.4%, and average specificity of 59.1%; *p* < 0.001), but a slightly lower predictive ability for mortality at a cut-off value of 221 (AUC 0.7, Sn = 65.1%, Sp = 62.8%; *p* < 0.001).

**Figure 5:**
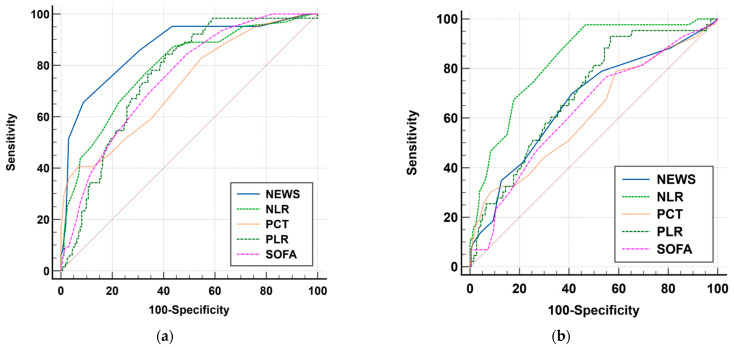
Comparison of receiver operating characteristic (ROC) representing the prognostic performance of the studied parameters (NLR, PLR, NEWS, SOFA, and PCT) for predicting septic shock (**a**) and urosepsis-related mortality (**b**). The dotted line indicates non-discrimination (AUC = 0.5).

The statistical evaluation of the prognostic performance of inflammatory markers included multiple ROC curve comparisons for the NEWS and SOFA scores as well as NLR, PLR, and PCT values. The ROC curves are displayed together on a single graph, and AUCs with standard errors (SEs) and 95% CIs were calculated to evaluate the discriminative performance of these parameters against the line of no discrimination (AUC 0.5) before applying DeLong’s statistical test for comparison (Figure 5). Table 4 presents a summary of the evaluated parameters’ performance results in the comparative ROC curve analysis. The AUC values, measured between 0.723 and 0.866, indicate different discriminatory capabilities.

The NLR showed very good diagnostic performance for the prediction of septic shock with an AUC of 0.789 (95% CI: 0.730–0.841), surpassing the discriminatory value of PLR (AUC: 0.759; 95% CI: 0.698–0.814), SOFA, and PCT. Although the NEWS score obtained the highest AUC value (0.866), the statistical comparison between NEWS and NLR failed to reveal a significant difference (*p* = 0.100); this means that the NLR can be considered as a comparable tool for identifying septic shock in patients. The PLR demonstrated acceptable discrimination power and maintained a statistically significant difference from NEWS (*p* < 0.05), thus making it a useful supplementary marker—particularly in resource-limited settings that lack dedicated laboratory markers for urosepsis. Regarding mortality, the NLR outperformed all other evaluated parameters and achieved the highest AUC value of 0.834 (95% CI: 0.778–0.880), which made it the most reliable indicator for prediction of mortality. The statistical analysis showed that the NLR outperformed the PLR (*p* < 0.01) and SOFA and PCT scores (*p* < 0.001), as well as the NEWS score (*p* < 0.01). The PLR obtained an AUC value of 0.700 (95% CI: 0.636–0.760), indicating a moderate prognostic capability, although it still outperformed the SOFA, PCT, and NEWS scores. These results demonstrate that the NLR may serve as a highly effective prognostic biomarker for the prediction of both clinical deterioration and urosepsis-related mortality. Overall, the NLR demonstrates strong predictive value for mortality risk assessment while the PLR exhibits moderate, yet meaningful performance. The obtained results indicate that these two basic haematological ratios have high potential as accessible biomarkers for early assessment of urosepsis.

Subsequently, we performed a multivariable binary logistic regression for predictors of septic shock and mortality (Table 5). The NLR was confirmed to be an independent predictor for both shock and mortality, as each unit increase in the NLR value was associated with 24% higher odds of septic shock (adjusted OR 1.24, 95% CI 1.15–1.35, *p* < 0.001) and 26% higher odds of in-hospital death (adjusted OR 1.26, 95% CI 1.16–1.37, *p* < 0.001). The PLR also retained a significant association, although its effect size was weaker in all cases and, so, its clinical impact is likely modest. Among the considered comorbidities, chronic kidney disease significantly increased the risk of septic shock, whereas diabetes mellitus and ischaemic heart disease were associated with lower odds. Regarding in-hospital mortality, dementia emerged as a significant independent risk factor, while chronic liver disease showed a strong trend toward significance. Taken together, these findings indicate that the NLR is a robust and independent prognostic marker of urosepsis, outperforming PLR and remaining significant even after controlling for clinical severity and major comorbidities. The associations between certain comorbidities and outcomes further highlight the importance of individualised risk assessments in this patient population.

## 4. Discussion

Urosepsis represents a distinct clinical and pathophysiological entity, differing from other forms of sepsis. The development of this condition occurs mainly through urinary tract obstruction, thus distinguishing it from pulmonary or intra-abdominal sepsis. It usually requires emergency decompression for source control (e.g., via ureteral stenting or percutaneous nephrostomy), but these procedures sometimes may paradoxically cause a sudden bacteremia and endotoxemia, resulting in abrupt septic shock [9,10].

The main pathogens in this context include Gram-negative bacilli, such as Escherichia coli and Klebsiella pneumoniae, that produce substantial amounts of lipopolysaccharides (LPSs). The inflammatory responses of neutrophils, monocytes, and endothelial cells to LPSs leads to elevated levels of pro-inflammatory cytokines and endothelial damage, ultimately resulting in multi-organ failure [11]. These pathophysiological mechanisms explain why biomarkers measuring leukocyte changes, such as the neutrophil-to-lymphocyte ratio (NLR), are particularly important in the context of urosepsis [11].

Analysis of the biomarkers in urosepsis patients has indicated elevated leukocyte counts, higher NLR, and strong systemic inflammatory responses when compared to other septic populations [12]. In a multi-centre study, Wagenlehner et al. revealed that patients with urosepsis presented with septic shock more frequently than pneumonia or abdominal infection patients, even though their comorbidity status was similar [13]. The mortality rate in urosepsis cases varies between 15 and 40% and depends heavily on both prompt antimicrobial treatment and immediate source management [14]. The clinical differences characterizing uroseptic patients require the development of straightforward biomarkers which allow for the rapid and accurate determination of patient risk levels. These observations support the independent evaluation of NLR and PLR as prognostic markers in urosepsis, because the combination of urinary obstruction with Gram-negative bacteraemia and sudden haemodynamic deterioration produces a distinct clinical problem when compared to other cases of sepsis [15].

Our prospective cohort study demonstrated that the NLR may serve as a strong independent factor for the prediction of both septic shock and in-hospital mortality, with their respective odds increasing by 21% and 25% for each unit increase in the NLR after controlling for age, sex, comorbidities, and severity scores. While the PLR remained statistically significant, its effect size was small, indicating its limited clinical utility as a standalone test. The risk of septic shock was more than three times higher in patients with chronic kidney disease than in those with other comorbidities, while cerebrovascular disease independently predicted mortality. Our research results align with those reported in our previous study, Dragoescu et al. (2024), which established that a NLR value above 10.8 correctly identified sepsis-associated delirium with 87% accuracy and an AUC of 0.77 [16].

Our research further supports previous studies showing that the NLR can serve as a useful prognostic indicator in sepsis patients. For example, Salciccioli et al. (2015) and Forget et al. (2017) showed that elevated NLR levels correlated with higher mortality rates among patients with sepsis [17,18]. Djordjevic et al. (2018) showed that NLR ≥ 10 had an AUC of 0.73 for predicting ICU mortality, comparable to the SOFA score [19]. Kaushik R et al. (2018) established that the NLR outperformed the PLR as a predictor of sepsis-related mortality [20]. Kriplani et al. (2022) demonstrated that NLR and PLR values measured at admission proved effective in predicting sepsis after percutaneous nephrolithotomy [21]. Karamouzos et al. (2022) showed that elevated NLR and PLR values predicted in-hospital death in septic patients [1]. Wang et al. (2022) performed a meta-analysis of 16 studies and verified that the PLR has a significant association with mortality [4].

Wu et al. (2023) demonstrated that the NLR achieved 97% specificity in detecting early post-percutaneous nephrolithotomy SIRS, performing better than PCT as a rule-in marker for infection [22]. Guliciuc et al. (2023) demonstrated that the NLR increased before SOFA detected organ dysfunction, thus making it suitable for early assessment of urosepsis in patients [23]. Mireștean et al. (2023) and Thombare et al. (2023) proved that using the NLR and PLR together with other biomarkers enhanced the predictive accuracy in septic patient groups [7,8]. Relevant research findings continued to grow in 2024: Li et al. demonstrated that the PLR and NLR successfully predicted urosepsis following ureteral stone surgery with PLR, achieving results similar to PCT [24]. Villanueva-Congote et al. (2024) established that elevated NLR and PLR values independently predicted ICU admission and vasopressor requirement in obstructive uropathy patients suspected of urosepsis [25]. Wu et al. (2024) established the NLR as a predictor of mortality in more than 10,000 patients, while Zhang et al. (2024) proved that the NLR and NPR provided better prognostic outcomes when considered together [2,26]. Islam et al. (2024) demonstrated that the NLR/PLR represent cost-effective solutions for emergency care worldwide [27].

Although the abovementioned research has provided strong evidence, not all studies have demonstrated identical results. Schupp et al. (2023) found that the NLR failed to predict 30-day mortality in bacteraemia patients when comorbidity burden was considered, obtaining an AUC value near 0.49 [28]. The differences between cut-off thresholds, infection sources, patient populations, and blood sampling times may explain this variability [28]. The specificities of the NLR and PLR become less reliable when patients have cancer, are receiving steroid therapy, or have autoimmune conditions [5]. The differences in these biomarkers in specific patient groups (including those with urosepsis) require further investigation.

Our ROC analysis demonstrated that the NLR performs better than the PLR. The AUC value for mortality prediction using the NLR reached 0.834, exceeding those for the PLR (0.700), SOFA (0.631), and PCT (0.618) scores. The NEWS score demonstrated better performance in predicting septic shock, with an AUC of 0.866, but failed to predict mortality. These findings align with those of Russell et al. (2019), who demonstrated that the NLR provides a cost-effective solution in resource-limited environments [29].

The PLR has shown promising results in certain studies (e.g., Wang et al. in 2022), but produces an equally divided assessment [4]. Shen et al. (2019) demonstrated that high PLR values were associated with poor survival outcomes in sepsis patients, but the relationship did not persist across different PLR threshold values [30]. The prognostic value of the PLR in sepsis continues to be evaluated through accumulating evidence. In a prospective Syrian cohort study, Al-Abdou and Al-Daher (2024) showed that elevated PLR levels at admission strongly predicted in-hospital death, with an AUC of 0.67 (95% CI 0.60–0.74), sensitivity of 60%, and specificity of 70% [31]. The prognostic value of PLR monitoring became evident through its sustained elevation in non-survivors during the first five days of hospitalisation, while survivors showed decreasing PLR levels [31]. The predictive power of the SOFA score and the NLR exceeded that of the PLR in our research, indicating that the PLR maintains statistical relationships but produces smaller effect sizes than the NLR. Zhang et al. (2024) recently investigated NLR_NPR as a predictive tool, which outperformed NLR in sepsis patient cohorts but requires larger patient populations [2]. Multiple factors contribute to these discrepancies, including the use of different cut-offs and measurement times for biomarkers, the inclusion of various patient groups and sepsis subtypes (e.g., urosepsis vs. pneumonia), and the inconsistent adjustment of key covariates such as comorbidities and age [2]. Our research provides a better understanding through its focus on urosepsis patients and its implementation of thorough multivariable statistical methods. The study results demonstrated that the NLR maintained its status as an independent risk factor, while the PLR presented a minimal effect size. These results indicate the consistent utility of the NLR in pathophysiologically uniform patient groups, while the PLR maintains a complex role in clinical practice.

The prognostic value of the PLR received additional evidence from Malah et al. (2024), who analysed 54 septic patients in an Egyptian ICU and showed that PLR > 229 predicted 28-day mortality with an AUC of 0.945, sensitivity of 86%, and specificity of 88% [32]. They demonstrated that PLR monitoring could be useful as survivors experienced a gradual decrease in PLR levels during the first week, while non-survivors maintained elevated PLR values. The authors noted that the PLR is inexpensive, universally available, and correlated with both the SOFA score and clinical outcomes such as ICU stay, even if the SOFA and PCT scores provided better predictive accuracy (AUCs of 0.982 and 0.977) [32]. The results of this study support our findings as the PLR maintained statistical significance but produced smaller effect sizes than the NLR, indicates its potential utility as an additional tool in prognostic models.

The patients in our cohort developed urosepsis from various sources, including urinary tract obstructions, catheter-associated infections, and post-surgical complications. We recognise that the inflammatory response and biomarker dynamics may differ between these subgroups. Our study was not powered to conduct a detailed subgroup analysis, but future multicenter studies stratifying by aetiology could clarify whether the predictive accuracy of NLR and PLR varies with the underlying source of infection.

The analysis becomes more robust with the inclusion of comorbidity data. One study found that septic shock was strongly linked to chronic kidney disease, as CKD patients have impaired immune clearance [33]. The presence of cerebrovascular disease independently led to higher mortality rates due to decreased physiological capacity. Meanwhile, the protective effects of diabetes mellitus and ischaemic heart disease on the development of septic shock might be explained by chronic inflammation and the protective effects of medications such as statins, ACE inhibitors, or beta-blockers, which can modulate inflammatory responses, as has been previously suggested [34]. Another possible reason is selection bias, since patients with advanced or unstable cardiovascular disease were excluded from our study. Consequently, these findings must be regarded with caution and confirmed in more extensive studies.

### 4.1. Clinical Implications

From a clinical standpoint, the neutrophil-to-lymphocyte ratio (NLR) is a rapid, inexpensive, and universally available biomarker that can be derived from a routine complete blood count (CBC) without additional cost or delay. It is useful in complementing established severity scores such as SOFA and NEWS, providing early bedside risk stratification before more complex laboratory results are available. Our findings suggest that an admission NLR ≥ 13 may be a practical threshold for identifying patients at increased risk of septic shock and in-hospital mortality. The NLR value of 13 was helpful for early risk stratification but requires external validation and should not yet be adopted in clinical protocols. This cut-off could be useful in the emergency department or in the initial ICU evaluation, alerting clinicians that the patient may benefit from early ICU referral or transfer for closer monitoring, aggressive haemodynamic optimisation and closer fluid/vasopressor titration, expedited source control procedures (e.g., drainage of urinary obstruction, removal of catheters, surgical debridement), timely escalation of antimicrobial therapy, and enhanced vigilance for organ dysfunction, even if baseline severity scores are not yet high.

In contrast, the platelet-to-lymphocyte ratio (PLR) appeared to be less robust as a standalone marker in our cohort. Nonetheless, its value should not be dismissed; in particular, the PLR may add incremental information when interpreted in conjunction with the NLR, particularly in patients with marked thrombocytosis or thrombocytopenia. Furthermore, Malah et al. and Al-Abdou & Al-Daher have shown that persistently elevated PLR over the first week correlates with poor outcomes, suggesting that serial PLR measurements could provide information on the trajectory of the disease. Finally, the PLR may be used as part of combined inflammatory ratios or multi-marker models (e.g., NLR + PLR + PCT) providing improved predictive accuracy, especially in heterogeneous sepsis populations [31,32].

The most useful application of the NLR and PLR may be in early triage and decision making, especially in resource-limited settings where advanced biomarkers (e.g., PCT, IL-6) may not be readily available. Finally, the simplicity and low cost of obtaining NLR/PLR values make them particularly attractive for low- and middle-income countries, where access to advanced laboratory markers is limited. Thus, the incorporation of these ratios into sepsis care pathways could serve as a cost-effective strategy to improve the early recognition and management of sepsis globally.

### 4.2. Limitations

Multiple limitations of this study should be evaluated to understand the presented results properly. The research was conducted at one tertiary care centre, potentially preventing the generalisation of the findings to other healthcare facilities with different patient populations, antimicrobial resistance, resource capabilities, and sepsis management protocols. The research did not track essential variables such as time to source control, pathogen virulence, and antibiotic resistance patterns. The study measured NLR and PLR values at admission but did not track their changes during hospitalisation, which could have added prognostic value. The study included patients with different urosepsis aetiologies (obstructive vs. non-obstructive infections), which could have affected the biomarkers’ performance. The number of patients was insufficient to perform comprehensive evaluations of biomarkers performance based on different urosepsis aetiologies. The results of this research apply specifically to patients who meet the Sepsis-3 criteria for urosepsis and may not apply to those with other infection types or early sepsis cases without organ dysfunction. Additional research must be conducted in multiple centres to track biomarkers over time and develop standardised blood sampling protocols in order to confirm the presented results and determine the precise value of the NLR and PLR in urosepsis risk assessment.

## 5. Conclusions

The NLR and PLR are now recognized in the medical field as important biomarkers which can facilitate the diagnosis of urosepsis. The NLR is as an inexpensive independent biomarker which was found to predict septic shock and in-hospital mortality in patients with urosepsis. The identification of high-risk patients who require ICU admission, close monitoring, and rapid source control can be achieved with an NLR threshold of ≥13. Although the PLR demonstrated weaker predictive power, its results can enhance the diagnostic process when used alongside other diagnostic markers. The NLR and PLR are reliable and non-inferior markers for the management of urosepsis, facilitating the early detection of serious infection and the prediction of septic shock.

## Figures and Tables

**Table 1:** Comparison of demographic data, biological parameters, and scores between patients with urosepsis and those with septic shock.

Parameter	Total (*n* = 223)	Urosepsis (*n* = 159)	Septic Shock (*n* = 64)	*p*
Age (years)	63.9 ± 13.0	63.4 ± 13.9	65.1 ±10.3	0.156 *
Sex (M/F)	141/82	102/57	39/25	0.652 #
SOFA score	6 [4–8]	5 [4–7]	8 [6–10]	<0.001 ^
NEWS	6 [5–8]	5 [5–7]	9 [7–10]	<0.001 ^
GCS	12 [11–14]	13 [12–14]	11 [10–12]	<0.001 ^
MAP (mmHg)	74.2 ± 9.6	78.3 ± 7.7	64.1 ± 5.3	<0.001 *
HR (beats/min)	86 ± 19	81 ± 12	98 ± 27	<0.001 *
RR (breaths/min)	23 ± 7	22 ± 6	27 ± 8	<0.001 *
WBC (×10^3^/mm^3^)	16 [14–19]	16 [13–19]	16 [14–18]	0.562 ^
NEU (×10^3^/mm^3^)	13 [11–16]	13 [11–16]	13 [11–16]	0.423 ^
LYM (×10^3^/mm^3^)	1.2 [0.8–1.6]	1.3 [0.8–1.5]	1.0 [0.6–1.8]	0.496 ^
PLT (×10^3^/mm^3^)	203 [160–275]	216 [162–277]	195 [156–237]	0.112 ^
Lactate (mmol/L)	1.3 [1.0–1.9]	1.2 [1.0–1.7]	1.9 [1.3–3.9]	<0.001 ^
Creatinine (mg/dL)	1.4 [1.0–2.3]	1.2 [1.0–2.2]	2.0 [1.4–2.3]	<0.05 ^
Bilirubin (mg/dL)	1.5 [1.1–2.2]	1.3 [1.1–2.1]	2.2 [1.3–2.8]	<0.05 ^
NLR	12 [9–15]	11 [8–13]	15 [13–20]	<0.001 ^
PLR	198 [144–277]	173 [133–243]	268 [207–329]	<0.001 ^
C-Reactive Protein (mg/L)	121 [96–143]	116 [94–135]	131 [101–150]	0.413 ^
Procalcitonin (ng/mL)	11.8 [9.1–14.6]	11.4 [8.5–13.5]	13.5 [11.5–19.2]	<0.001 ^

SOFA—Sequential Organ Failure Assessment; NEWS—National Early Warning Score; GCS—Glasgow Coma Scale; MAP—mean arterial pressure; HR—heart rate; RR—respiratory rate; WBC—white blood cells; NEU—neutrophils; LYM—lymphocytes; NLR—neutrophil-to-lymphocyte ratio; PLR—platelet-to-lymphocyte ratio; PLT—platelet; ESR—erythrocyte sedimentation rate. Data presented as mean and standard deviation, ratio, or median and interquartile range depending on data type and distribution (statistical tests: * = Student’s *t*-test, # = Chi-square test, ^ = Mann–Whitney U-test).

**Table 2:** Distribution of major comorbidities among patients with urosepsis and septic shock. Prevalence comparison performed via the Chi-square test.

Comorbidity	Total (*n* = 223)	Urosepsis (*n* = 159)	Septic Shock (*n* = 64)	*p*
Diabetes mellitus	72	46	26	0.312
Chronic kidney disease	56	36	20	0.221
Ischemic heart disease	48	31	17	0.415
Chronic obstructive pulmonary disease	34	22	12	0.540
Chronic liver disease	12	7	5	0.284
Dementia	10	6	4	0.442
Cerebrovascular disease	18	13	5	0.622
Malignancy (prior or active)	29	18	11	0.187

**Table 3:** Comparison of demographic data, biological parameters, and scores between survivors and non-survivors.

Parameter	Total (*n* = 223)	Survivors (*n* = 180)	Deceased (*n* = 43)	*p*
Age (years)	63.9 ± 13.0	64.1 ± 13.2	62.8 ± 12.1	0.253 *
Sex (M/F)	141/82	119/61	22/21	0.652 #
SOFA score	6 [4–8]	6 [4–8]	7 [6–9]	<0.01 ^
NEWS	6 [5–8]	6 [5–7]	7 [6–10]	<0.001 ^
GCS	12 [11–14]	13 [11–14]	12 [10–13]	<0.05 ^
MAP (mmHg)	74.2 ± 9.6	75.1 ± 9.1	70.3 ± 10.7	<0.01 *
HR (beats/min)	86 ± 19	86 ± 17	88 ± 28	0.325 *
RR (breaths/min)	23 ± 7	23 ± 6	26 ± 9	<0.05 *
WBC (×10^3^/mm^3^)	16 [14–19]	16 [13–18]	17 [14–22]	<0.05 ^
NEU (×10^3^/mm^3^)	13 [11–16]	13 [11–15]	15 [11–17]	<0.05 ^
LYM (×10^3^/mm^3^)	1.2 [0.8–1.6]	1.1 [0.6–1.4]	1.2 [0.7–1.6]	0.231 ^
PLT (×10^3^/mm^3^)	203 [160–275]	208 [164–275]	195 [155–266]	0.453 ^
Lactate (mmol/L)	1.3 [1.0–1.9]	1.3 [1.0–1.9]	1.4 [1.2–2.4]	0.373 ^
Creatinine (mg/dL)	1.4 [1.0–2.3]	1.3 [1.0–2.5]	1.4 [1.1–2.0]	0.610 ^
Bilirubin (mg/dL)	1.5 [1.1–2.2]	1.8 [1.1–2.3]	1.2 [1.0–2.2]	<0.05 ^
NLR	12 [9–15]	11 [8–14]	16 [13–22]	<0.001 ^
PLR	198 [144–277]	181 [137–265]	265 [197–347]	<0.001 ^
C-Reactive Protein (mg/L)	121 [96–143]	120 [96–140]	126 [95–151]	0.342 ^
Procalcitonin (ng/mL)	11.8 [9.1–14.6]	11.7 [8.8–14.3]	12.6 [10.6–18.4]	<0.05 ^

SOFA—Sequential Organ Failure Assessment; NEWS—National Early Warning Score; GCS—Glasgow Coma Scale; MAP—mean arterial pressure; HR—heart rate; RR—respiratory rate; WBC—white blood cells; NEU—neutrophils; LYM—lymphocytes; NLR—neutrophil-to-lymphocyte ratio; PLR—platelet-to-lymphocyte ratio; PLT—platelet; ESR—erythrocyte sedimentation rate. Data presented as mean and standard deviation, ratio, or median and interquartile range depending on data type and distribution (statistical tests: * = Student’s *t*-test, # = Chi-square test, ^ = Mann–Whitney U-test).

**Table 4:** Comparison of ROC curve performance among the studied parameters, showing the area under the curve (AUC) value, standard error (SE), and 95% confidence interval (CI) for each parameter. Higher AUC values indicate better discriminatory power. DeLong’s statistical test was used for comparisons between multiple AUCs.

	Urosepsis vs. Septic Shock			Survivors vs. Deceased		
Variable	AUC	SE	95% CI	AUC	SE	95% CI
NEWS	0.866	0.0281	0.814 to 0.908	0.666	0.0473	0.600 to 0.727
SOFA	0.748	0.0337	0.686 to 0.804	0.631	0.0475	0.564 to 0.695
PCT	0.723	0.0381	0.660 to 0.781	0.618	0.0504	0.551 to 0.682
PLR	0.759	0.0328	0.698 to 0.814	0.700	0.0423	0.636 to 0.760
NLR	0.789	0.0340	0.730 to 0.841	0.834	0.0317	0.778 to 0.880

**Table 5:** Multivariable binary logistic regression analysis for the prediction of severity (shock) and outcome (death). Results are presented as adjusted odds ratios (OR) with 95% confidence intervals (CI). *p*-values were calculated using the Wald test. COPD = chronic obstructive pulmonary disease.

Urosepsis (n = 223)	Severity—Sepsis Shock			Outcome—Death		
Variable	Adjusted OR	95% CI	*p*	Adjusted OR	95% CI	*p*
NLR	1.24	1.15–1.35	<0.001	1.26	1.16–1.37	<0.001
PLR	1.06	1.01–1.19	0.043	1.09	1.03–1.25	0.027
Diabetes mellitus	0.38	0.16–0.92	0.032	1.54	0.63–3.77	0.348
Chronic kidney disease	3.39	1.48–7.74	0.004	0.83	0.33–2.10	0.701
Ischemic heart disease	0.27	0.09–0.82	0.021	1.09	0.34–3.52	0.884
COPD	0.72	0.26–2.00	0.525	1.10	0.34–3.52	0.871
Chronic liver disease	0.49	0.09–2.66	0.407	4.25	0.90–20.07	0.068
Dementia	0.27	0.06–1.17	0.080	4.32	1.31–14.21	0.016
Cerebrovascular disease	0.74	0.12–4.74	0.752	2.02	0.39–10.56	0.406
Malignancy (prior/active)	0.55	0.16–1.98	0.363	2.09	0.52–8.37	0.300

## Data Availability

The data from this study is not available to the public because of ethical and privacy regulations. The corresponding author can provide access to anonymized datasets after receiving a reasonable request and obtaining approval from our institutional ethics committee.

## Abbreviations

The following abbreviations are used in this manuscript:

AUC	Area Under the Curve
CBC	Complete Blood Count
CI	Confidence Interval
COPD	Chronic obstructive pulmonary disease
CRP	C-Reactive Protein
CT	Computed Tomography
ECLIA	Electro-Chemiluminescence Immunoassay
GCS	Glasgow Coma Scale
HR	Heart Rate
ICU	Intensive Care Unit
IQR	Interquartile Range
LYM	Lymphocytes
MAP	Mean Arterial Pressure
NEU	Neutrophils
NEWS	National Early Warning Score
NLR	Neutrophil-to-Lymphocyte Ratio
PCT	Procalcitonin
PLR	Platelet-to-Lymphocyte Ratio
PLT	Platelets
ROC	Receiver Operating Characteristic
SD	Standard Deviation
SE	Standard Error
SIRS	Systemic Inflammatory Response Syndrome
SOFA	Sequential Organ Failure Assessment
UTI	Urinary Tract Infection
WBC	White Blood Cell Count
